# Morphology, Molecular Genetics, and Bioacoustics Support Two New Sympatric *Xenophrys* Toads (Amphibia: Anura: Megophryidae) in Southeast China

**DOI:** 10.1371/journal.pone.0093075

**Published:** 2014-04-08

**Authors:** Yingyong Wang, Jian Zhao, Jianhuan Yang, Zhixin Zhou, Guoling Chen, Yang Liu

**Affiliations:** 1 State Key Laboratory of Biocontrol, School of Life Sciences, Sun Yat-sen University, Guangzhou, China; 2 Kadoorie Conservation China, Kadoorie Farm and Botanic Garden, Hong Kong, China; 3 Key Laboratory of Animal Ecology and Conservation Biology, Institute of Zoology, Chinese Academy of Sciences, Beijing, China; University of Guelph, Canada

## Abstract

Given their recent worldwide declines and extinctions, characterization of species-level diversity is of critical importance for large-scale biodiversity assessments and conservation of amphibians. This task is made difficult by the existence of cryptic species complexes, species groups comprising closely related and morphologically analogous species. The combination of morphology, genetic, and bioacoustic analyses permits robust and accurate species identification. Using these methods, we discovered two undescribed *Xenophrys* species, namely *Xenophrys lini* **sp. nov.** and *Xenophrys cheni* **sp. nov.** from the middle range of Luoxiao Mountains, southeast China. These two new species can be reliably distinguished from other known congeners by morphological and morphometric differences, distinctness in male advertisement calls, and substantial genetic distances (>3.6%) based on the mitochondrial 16s and 12s rRNA genes. The two new species, together with *X. jinggangensis*, are sympatric in the middle range of Luoxiao Mountains but may be isolated altitudinally and ecologically. Our study provides a first step to help resolve previously unrecognized cryptic biodiversity and provides insights into the understanding of *Xenophrys* diversification in the mountain complexes of southeast China.

## Introduction

Accurate taxonomic recognition is a prerequisite for preserving amphibian biodiversity, especially in the context of amphibian declines and extinctions worldwide [1]. This fundamental task is challenged by the existence of cryptic species complexes [2], a group consisting of two or more species that are reproductively isolated from each other but virtually identical in morphology [3]. Frogs and other groups of Amphibians are known to harbor substantially underestimated cryptic species diversity [4]. Hence unambiguous species delineation may be difficult in some frog groups based on morphological characteristics exclusively [5], but it is very important to provide a solid basis for conservation management, as well as deeper understanding of the macro-evolutionary patterns in amphibians [6].

The horned toads, *Megophrys* Kuhl & Van Hasselt, 1822 and *Xenophrys* Günther, 1864, in the family Megophryidae, are an exemplary group with high cryptic species diversity [7]–[10], making their systematics and taxonomies poorly understood and considerablly debated, even though herpetologists have employed various taxonomic methods [8], [11]–[14]. Pending comprehensive phylogenetic and morphological research, we followed the recommendations of Li & Wang [13] and Pyron and Wiens [15] that *Xenophrys* is distinguished from *Megophrys* and all previously known *Megophrys* species in China and should be assigned to the genus *Xenophrys*. Currently, the genus *Xenophrys* contains 46 species and is distributed in Southeast Asia from the southern and eastern Himalayan regions to Borneo [16]. There are 31 species of *Xenophrys* recognized from China, among which only five have a body length less than 50 mm in both males and females in southeast China [10]. Species in this group include *X. boettgeri* and *X. kuatunensis* in the Wuyi Mountains, *X. huangshanensis* in the Huangshan Mountains, and the recently described *X. jinggangensis* from Mount Jinggang (26°13′–26°52′N, 113°59′–114°18′E), situated in the border between the Jiangxi and Hunan provinces [10]. Noticeably, three new species of Megophryid toads described from northeast India very recently [9]. Together with *X. jinggangensis*, these discoveries raise the possibility of new cryptic species that might be discovered in Southeast Asia and China. However, due to the morphological similarity of Megophryid toads, only morphological characters may not be sufficient to diagnose taxonomies. To implement unbiased species delineation in cryptic amphibians, integrating evidence from morphology, DNA sequence data, and behavior may be critically necessary [17].

In particular, molecular genetic approaches enable us to decipher phylogenetic relationships and thus the evolutionary history of a large number of species, thereby solving taxonomic uncertainties [18]. In addition, bioacoustic analysis is a very useful approach in species diagnosis because many male frogs and toads have species-specific advertising vocalization during breeding seasons [19]. Like birds, these sounds are courtship signals used for mate choice by females and male-male competition, and thus presents an important behavioral pre-mating reproductive barrier under sexual selection [20]. Indeed, combined multiple lines of evidence from different methods assist in discovering unrecognized amphibian species regularly and this substantially inflates amphibian species diversity across the world, e.g. [21]–[24].

To understand the distribution and ecology of the newly described *X. jinggangensis*, we carried out extensive herpetological surveys during 2011–2013 around the middle range of Luoxiao Mountains, SE China. Interestingly, we also found two small and unknown Megophryid toads. Both of these species are small in body length (<45 mm), they can be assigned to the genus *Xenophrys* based on the following characteristics: head broad and depressed, tympanum distinct, tubercles on the outer edge of the upper eyelids short, tubercles on the snout absent, no mid-dorsal fold, no black horny spines on dorsum, hind limbs long, and heels overlap [13]. Furthermore, we also noticed that the vocalizations and phylogenetic relationships of these two unknown *Xenophrys* toads seemed to be distinct from that of *X. jinggangensis*. Thus, we conducted morphological, bioacoustics, and molecular genetic analyses to resolve the taxonomic status and affinities of these two taxa. Based on all these evidence, we describe two new species from southeast China.

## Materials and Methods

### Ethics Statement

Permissions to visit the study sites were issued by the management administration of the reserves. We obtained permissions for specimen and tissue collection from Jiangxi Provincial Forestry Bureau. This study did not involve endangered or protected species. All the animal operations were approved by the Institutional Ethical Committee of Animal Experimentation of Sun Yat-sen University and strictly complied with the ethical conditions by the Chinese Animal Welfare Act (20090606).

### DNA sample collection

**Figure 1:**
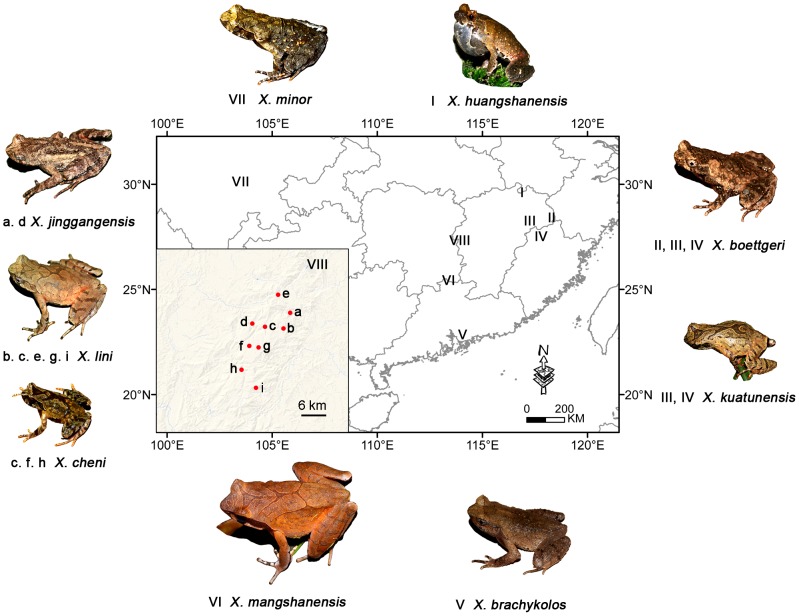
Sampling localities of *Xenophrys* toads in southern China. These include: **I**. Huangshan Mountains, here collected refer to *X. huangshanensis* from Mt. Dazhang, Wuyuan, Jiangxi; **II**. *X. boettgeri* from Mt. Tongbo, Jiangxi; **III**. *X. boettgeri* from Mt. Yangjifeng, Jiangxi; **IV**. *X. kuatunensis* from Guadun ( = Kuatun), Fujian; **V**. *X. brachykolos* from Hong Kong; **VI**. *X. mangshanensis* from Mt. Nanling, Guangdong; **VII**. *X. minor* from Mt. Emei and Mt. Laojun, Sichuan; **VIII**. the middle Luoxiao Mountains: *X. jinggangensis* from the peak of Mt. Jinggang, Jiangxi (**a**), and Taoyuandong, Hunan (**d**); *Xenophrys lini* **sp. nov.** from Dabali (**b**) and Niushiping (**g**) **in** Hunan; Jingzhushan (**c**), Bamianshan (**e**), Nafengmian (**i**) in Jiangxi; *Xenophrys cheni* **sp. nov.** from Jingzhushan (**c**), Jiangxi, Dayuan (**f**) and Lishuzhou (**h**) in Hunan.

To reconstruct the phylogenetic relationships among *Xenophrys* species in southern China, we collected samples of *X. jinggangensis*, *X. cheni* **sp. nov.**, and *X. lini* **sp. nov.** from the middle range of Luoxiao Mountains, situated in the border between the Jiangxi and Hunan Provinces, *X. brachykolos* from Hong Kong, *X. boettgeri* from Mt. Yangjifeng and Mt. Tongbo, Jiangxi Province, *X. kuatunensis* from Guadun (Kuatun) Village, Fujian Province, situated in the Wuyi Mountains, *X. huangshanensis* from Wuyuan County, Jiangxi Province, situated in the Huangshan Mountains, *X. mangshanensis* from Mt. Nanling, Guangdong Province, and *X. minor* from central Sichuan province (Figure 1). An additional 16s rRNA sequence of *X. minor* deposited in GenBank was incorporated into our dataset. The data of all voucher specimens of the above species are shown in Table 1. All muscle tissue was preserved in 95% ethanol and stored in −80°C.

**Table 1:** Sampling locations, voucher data, and associated GenBank Accession numbers of *Xenophrys* toads in this study.

ID	Species	Locality	Voucher	GenBank Accession No.	
				16S rRNA	12S rRNA
1	*Megophrys nasuta*	Tenom Dist, Sabah, Malysia	FMNH 236525	DQ283342	DQ283342
2	*Paramegophrys oshanensis*	Sichuan, China	/	KC460337	KC460337
3			SYS a000496	JX867335	/
4			SYS a001564	KJ560371	/
5	*Xenophrys mangshanensis* (6)	Mt.Nanling, Guangdong, China	SYS a002025	KJ560372	/
6			SYS a002026	KJ560373	/
7			SYS a002118	KJ560374	/
8		Jiulianshan, Jiangxi, China	SYS a002119	KJ560375	/
9			SYS a001579	KJ560376	/
10	*X. kuatunensis* (3)	Mt. Wuyi, Fujian, Chjna	SYS a001590	KJ560377	/
11			SYS a001592	KJ560378	/
12		Mt. Yangjifeng, Jiangxi, China	SYS a000378	KJ560379	/
13			SYS a001671	JX867340	/
14	*X. boettgeri* (5)		SYS a001673	KJ560380	KJ560417
15		Mt. Tongba, Jiangxi, China	SYS a001683	KJ560381	KJ560418
16			SYS a001700	KJ560382	KJ560419
17		Wuyuan, Jiangxi, China	SYS a001322	KJ560383	/
18	*X. huangshanensis* (3)		SYS a001622	KJ560384	/
19			SYS a001623	KJ560385	/
20			CIB ZYC1500	AY561308	/
21		Emeishan, Sichuan, China	SYS a001804	KJ560386	KJ560420
22	*X. minor* (6)		SYS a001805	KJ560387	KJ560421
23			SYS a002164	KJ560388	KJ560422
24		Laojunshan, Sichuan, China	SYS a002165	KJ560389	KJ560423
25			SYS a002166	KJ560390	KJ560424
26			SYS a001427	KJ560391	/
27			SYS a001428	KJ560392	/
28		Jianggangshan, Jiangxi, China	SYS a001429	KJ560393	/
29	*X. cheni sp. nov.* (8)		SYS a001871*	KJ560394	KJ560425
30			SYS a001872*	KJ560395	KJ560426
31			SYS a002123*	KJ560396	KJ560427
32		Taoyuandong, Hunan, China	SYS a002124*	KJ560397	KJ560428
33			SYS a002142*	KJ560398	KJ560429
34		Jinggangshan, Jiangxi, China	SYS a001414	JX867338	/
35			SYS a001416	JX867337	/
36	*X. jinggangensis* (6)		SYS a001859	KJ560399	KJ560430
37		Taoyuandong, Hunan, China	SYS a001860	KJ560400	KJ560431
38			SYS a002131	KJ560401	KJ560432
39			SYS a002132	KJ560402	KJ560433
40			SYS a001502	JX867339	/
41	*X. brachykolos* (3)	Hong Kong, China	SYS a002258	KJ560403	KJ560434
42			SYS a002259	KJ560404	KJ560435
43			SYS a001420#	KJ560405	/
44			SYS a001421*	KJ560406	/
45			SYS a001422	KJ560407	/
46		Jinggangshan, Jiangxi, China	SYS a001489	KJ560408	/
47			SYS a001490	KJ560409	/
48	*X. lini sp.nov.*(12)		SYS a001491	KJ560410	/
49			SYS a002380*	KJ560411	KJ560436
50		Suichuan, Jiangxi, China	SYS a002370*	KJ560412	KJ560437
51			SYS a002371*	KJ560413	KJ560438
52		Baimianshan, Jiangxi, China	SYS a002382*	KJ560414	KJ560439
53			SYS a002383*	KJ560415	KJ560440
54		Taoyuandong, Hunan, China	SYS a002128*	KJ560416	KJ560441

Collection abbreviations: **CIB**, Chengdu Institute of Biology, the Chinese Academy of Sciences, China; **FMNH**, The Field Museum of Natural History, Chicago, USA; **SYS**, The Biology Museum, Sun Yat-sen University, China. The symbol ‘#’ with a voucher number indicates holotype and ‘*’ refers to paratype.

### DNA extraction and sequencing

Genomic DNA was extracted from the muscle tissue using a standard phenol-chloroform extraction protocol [25]. We amplified a fragment of the mitochondrial 16s rRNA gene from *Xenophrys* species using the primer pairs L3975 and H4551 [26], and mitochondrial 12s rRNA gene using the primer pair Fphe40L and 12S600H [27]. PCR amplifications were performed in a reaction volume of 25 µl containing 100 ng of template DNA, 0.3 mM of each PCR primer and 10 µl Premix EX Taq™ (Takara, Dalian, China). The PCR condition for 16s rRNA was, an initial denaturation step at 94°C for 1.5 min; 33 cycles of denaturation at 94°C for 45 s, annealing at 55°C for 45 s, and extending at 72°C for 90 s, and a final extension step of 72°C for 10 min. The PCR condition for 12s rRNA was, an initial denaturation step at 96°C for 2 min; 35 cycles of denaturation at 94°C for 15 s, annealing at 55°C for 1 min, and extending at 72°C for 1 min, and a final extension step of 72°C for 10 min. PCR products were purified with the GenElute™ PCR clean-up kit (Sigma-Aldrich, Dorset, UK). The purified products were sequenced with both forward and reverse primers using a BigDye Terminator v 3.1 Cycle Sequencing Kit (Applied Biosystems, Carlsbad, CA, USA) according to the manufacturer's instructions. The products were sequenced on an ABI Prism 3730 automated DNA sequencer (Applied Biosystems) in the Beijing Genomics Institute. All sequences have been deposited in GenBank (Table 1).

### Phylogenetic analyses

The resulting sequences were first aligned using the Clustal W algorithm [28] in BioEdit 7.0 [29], with default parameters and the alignment being checked and manually revised, if necessary. The degree of polymorphism of our sequences was assessed using DnaSP 5.10.1 [30]. The General Time-Reversible model [31] assuming a gamma-shaped distribution across sites [32] for both 16s rRNA and 12s rRNA were selected as the best-fit nucleotide substitution model using Akaike's Information Criterion [33] in jModelTest 1.0 [34]. The aligned sequences of 16s rRNA and 12s rRNA dataset were analyzed separately, using both maximum likelihood (ML) implemented in PhyML [35] on the ATGC online server (http://www.atgc-montpellier.fr/), and Bayesian inference (BI) using MrBayes 3.12 [36]. For ML analysis, the bootstrap consensus tree inferred from 1000 replicates was used to estimate nodal supports of inferred relationships on phylogenetic trees. Branches corresponding to partitions reproduced in less than 50% of the bootstrap replicates were collapsed. For the tree searching and optimization, we applied strategies described in Liang et al. [37]. For BI analysis, two independent runs, each comprising four Markov Chain Monte Carlo simulations were performed for two million iterations and sampled every 1000^th^ step. The first 25% of the samples were discarded as burn-in. Convergence of the Markov Chain Monte Carlo simulations was assessed by checking the average standard deviation of split frequencies between two runs using Tracer v.1.4 (http://tree.bio.ed.ac.uk/software/tracer/). We further applied ML and BI for the joint dataset of 16s and 12s rRNA using the above settings. For both ML and BI, the homologous sequences of *Paramegophrys oshanensis* and *Megophrys nasuta* from GenBank were chosen as outgroup (Table 1). Apart from phylogenetic tree-based methods, we also calculated ‘net between groups means distance’ between *Xenophrys* species at both genes using the mentioned nucleotide substitution models in MEGA 5.2 [38]. This method takes within-group variations (individual level) into account when calculating the average distances between taxa. Furthermore, to incorporate both 16s rRNA and 12s rRNA datasets, we generated Neighbor-Net (NN) networks [39] of *Xenophrys* samples using uncorrected *p*-distance as implemented in SplitsTree 4.10 [40].

### Bioacoustics analysis

We recorded the advertisement calls of male *Xenophrys* species (*X. jinggangensis*, *X. cheni* **sp. nov.**, *X. lini* **sp. nov.**, *X. boettgeri, X. huangshanensis*, and *X. kuatunensis*) by SONY ICD-MX20 IC at our sampling localities in southern China during 2011–2012. The calls of these species were frequently heard from June to September, temperatures varied in a small range between 15–18°C during the sound sampling period. Stereotypical male calls, which lasted between 20 s and 2 min, of each species were recorded in SONY MSV format and converted to 16-bit mono PCM format with resampling of 22 kHz. The spectrograms of male calls were generated in the Avisoft-SAS lab Lite software. We define a continuous vocalization with a pause less than 1 second as a call, and define the smallest non-split syllable as a note. The duration and frequency parameters of vocalization, such as call duration, notes per call and per second, note duration, inter-note interval, high and low frequency and frequency band-width were measured from the spectrograms. Differences between these *Xenophrys* species were tested with one-way ANOVA and Kruskal–Wallis tests in IBM SPSS Statistics 21.

### Morphological analysis

Specimens for morphometric analysis were fixed in 10% buffered formalin and then transferred to 70% ethanol for long-term preservation in The Museum of Biology, Sun Yat-sen University (SYS), Guangzhou, Guangdong Province, China. Measurements were made with digital calipers (Neiko 01407A Stainless Steel 6-Inch Digital Caliper, USA) to the nearest 0.1 mm. Abbreviations used are SVL = snout–vent length; HDL = head length, from the tip of the snout to the articulation of the jaw; HDW = head width, between left and right articulations of the quadratojugal and maxilla; SNT = snout length, from the tip the of snout to the anterior corner of the eye; EYE = eye diameter, from the anterior to the posterior corner of the eye; IND = internasal distance; IOD = interorbital distance; TMP = tympanum diameter; TEY = tympanum–eye distance, from the anterior edge of the tympanum to the posterior corner of the eye; HND = hand length, from the distal end of the radioulna to the tip of the distal phalanx of III; RAD = radioulna length; FTL = foot length, from the distal end of the tibia to the tip of the distal phalanx of III; TIB = tibia length; TaL = tail length in tadpoles, from the tip of the tail fin to the vent. Differences between these parameters in the two new species (males only) were further analyzed with Mann–Whitney U test in IBM SPSS Statistics 21.

### Nomenclatural Acts

The electronic edition of this article conforms to the requirements of the amended International Code of Zoological Nomenclature, and hence the new names contained herein are available under that Code from the electronic edition of this article. This published work and the nomenclatural acts it contains have been registered in ZooBank, the online registration system for the ICZN. The ZooBank LSIDs (Life Science Identifiers) can be resolved and the associated information viewed through any standard web browser by appending the LSID to the prefix “http://zoobank.org/”. The LSID for this publication is: urn:lsid:zoobank.orgub:C4999824-619C-4E25-B9B2-8E21B95A9C35. The electronic edition of this work was published in a journal with an ISSN, and has been archived and is available from the following digital repositories: PubMed Central, LOCKSS.

## Results

### Molecular phylogenetic analyses

**Figure 2:**
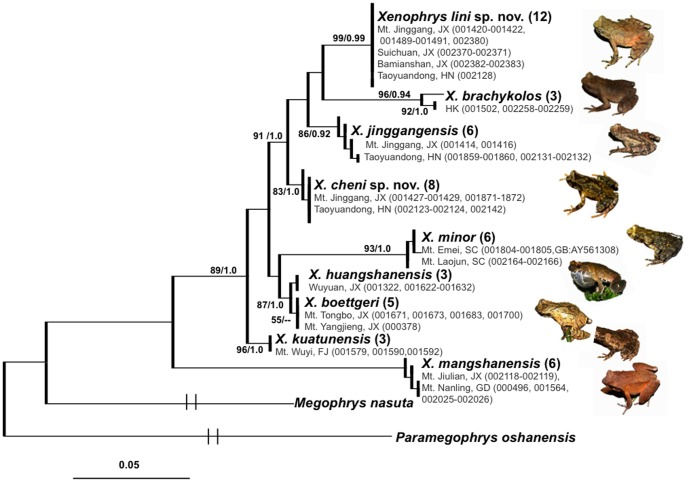
Bayesian inference and maximum-likelihood phylogenies. The species *Paramegophrys oshanensis* and *Megophrys nasuta* were included as outgroup. Numbers above or below branches are bootstrap values based on 1000 replicates for maximum-likelihood analyses (left, >50 retained) and Bayesian posterior probabilities (right, >0.5 retained). Vouchers and Genbank accessions are provided corresponding to information in Table 1. Abbreviations: FJ = Fujian; GD = Guangdong, HK = Hong Kong; HN = Hunan; JX = Jiangxi; SC = Sichuan.

**Figure 3:**
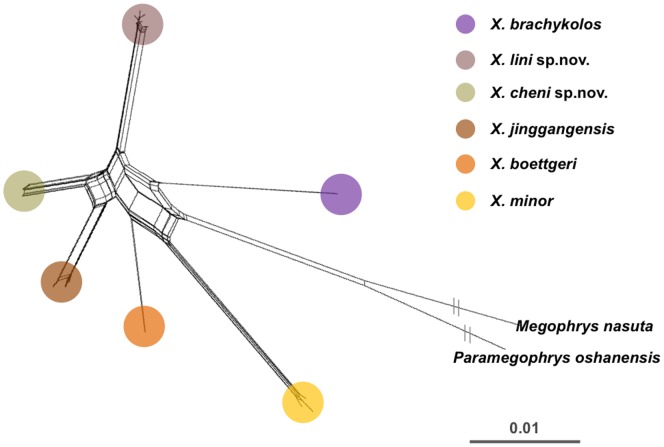
Multilocus mtDNA networks inferred by SplitsTree , The networks were constructed based on a concatenated dataset of 422 bp 16S rRNA and 498 bp 12S rRNA sequences for 25 *Xenophrys* individuals including *Paramegophrys oshanensis* and *Megophrys nasuta* as outgroup.

While we obtained a 422 bp of mitochondrial 16s rRNA sequence alignment from 52 *Xenophrys* samples, and we got a 498 bp of mitochondrial 12s rRNA sequence alignment from a smaller number of samples (N = 25) due to the lack of DNA or amplification difficulties (Table 1). The 16s rRNA alignment yielded 115 variable sites and 98 were parsimony-informative with five insertions. The 12s rRNA yielded 84 variable sites and 74 were parsimony-informative with four insertions. Indels were removed before phylogenetic analyses. For 16s rRNA, the ML and BI phylogenetic approaches resulted in virtually identical topology and all terminal clades had relatively high-supporting values. Both bootstrap supports and posterior probabilities for the clades representing *X. cheni* **sp. nov.** and *X. lini* **sp. nov.** were high (>80% for bootstrap proportions and 1.0 for Bayesian posterior probability, respectively; Figure 2). The *X. cheni* **sp. nov.**, *X. lini* **sp. nov.**, *X. jinggangensis*, and *X. brachykolos* might form a clade. The large-bodied *X. mangshanensis* was basal to the rest of the eight small-size congeners on the phylogenetic tree and showed large net average distances to others (12%–16%, Table 2). Phylogenetic analyses based on 12s rRNA also revealed well support for the clades representing the two new species (Figure S1), so as the results inferred by ML and BI based on the joint dataset of two mitochondrial locus (Figure S2). The net average genetic distance between the two new species was 3.6% (16s rRNA) and 5.7% (12s rRNA), respectively (Table 2). This differentiation was comparable to the divergence between *X. jingangensis* to *X. brachykolos*. Interestingly, the net average genetic distance between *X. boettgeri* and *X. huangshanensis* was only 0.005, in contrast to the values between the remaining comparisons (Table 2). Furthermore, in consistent with the phylogenetic gene trees, the inferred multi-locus network based on concatenated sequences of 16s and 12s rRNA (910 bp) strongly supported the genetic distinctness of *X. cheni* **sp. nov.** and *X. lini* **sp. nov.** (Figure 3).

**Table 2:** Net average genetic distances between *Xenophrys* species in southeast China.

	*Xma*	*Xku*	*Xhu*	*Xmi*	*Xbo*	*Xji*	*Xbr*	*Xch*	*Xli*
*X. mangshanensis*	-	NA	NA	NA	NA	NA	NA	NA	NA
*X. kuatunensis*	0.113±0.022	-	NA	NA	NA	NA	NA	NA	NA
*X. huangshanensis*	0.130±0.025	0.030±0.009	-	NA	NA	NA	NA	NA	NA
*X. minor*	0.165±0.032	0.063±0.016	0.056±0.014	-	0.110±0.028	0.090±0.022	0.140±0.034	0.107±0.026	0.145±0.036
*X. boettgeri*	0.121±0.023	0.030±0.009	0.005±0.004	0.052±0.013	-	0.064±0.017	0.120±0.032	0.067±0.018	0.098±0.026
*X. jinggangensis*	0.132±0.025	0.045±0.012	0.040±0.011	0.069±0.016	0.040±0.011	-	0.085±0.023	0.036±0.011	0.080±0.022
*X. brachykolos*	0.161±0.031	0.052±0.013	0.062±0.016	0.086±0.020	0.062±0.016	0.059±0.015	-	0.098±0.028	0.124±0.033
*X. cheni* **sp.nov.**	0.124±0.025	0.027±0.009	0.029±0.009	0.060±0.015	0.029±0.009	0.026±0.008	0.053±0.014	-	0.057±0.016
*X. lini* **sp.nov.**	0.134±0.026	0.042±0.012	0.046±0.012	0.085±0.019	0.046±0.012	0.040±0.011	0.054±0.014	0.036±0.011	-

The genetic distances, using the nucleotide substitution models determined by model tests mentioned in the text. The average distances and the associated standard errors are given based on 422 bp of mitochondrial 16s rRNA from 52 *Xenophrys* samples (below the diagonal) and 498 bp of mitochondrial 12s rRNA from 25 *Xenophrys* samples (above the diagonal).

### Acoustic analysis of advertisement calls

We recorded the male calls of 13 individuals from three sympatric *Xenophrys* species in Mt. Jinggang, *X. jinggangensis*, *X. cheni* **sp. nov.**, and *X. lini* **sp. nov.** (four, three, and six individuals, respectively). We also recorded the vocalizations of *X. kuatunensis* (N = 2), *X. huangshanensis* (N = 3), and *X. boettgeri* (N = 3) permitting additional comparisons (the typical calls of six analyzed *Xenophrys* species are available in Appendix S1).

**Figure 4:**
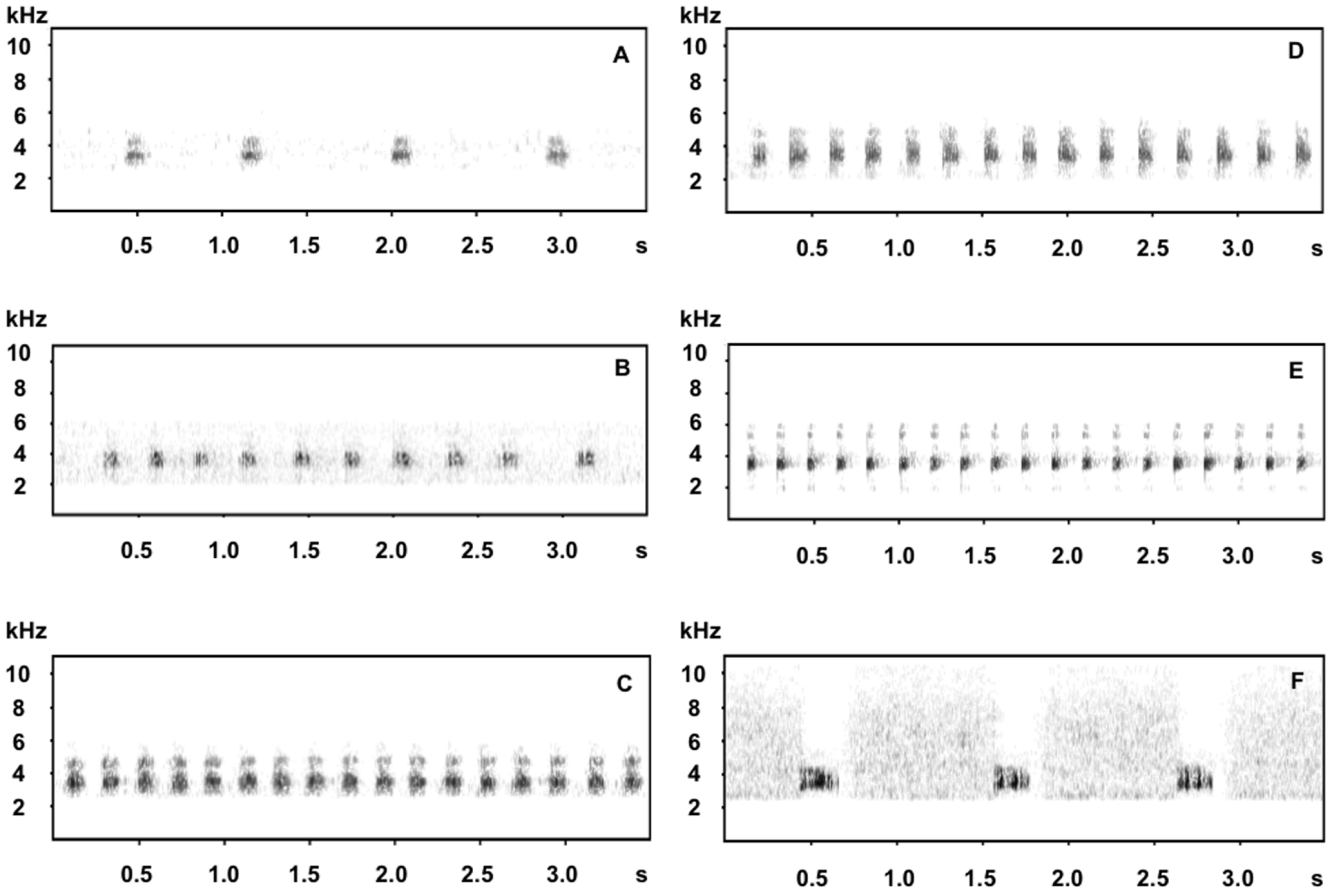
Typical spectrogram of male advertisement calls of six *Xenophrys* species. (A) *X. cheni* sp. nov., (B) *X. lini* sp. nov., (C) *X. jinggangensis*, (D) *X. huangshanensis*, (E) *X. boettgeri*, and (F) *X. kuatunensis*.

The male call of *X. cheni* **sp. nov.** has a much slower pace than *X. jinggangensis* and *X. lini* **sp. nov.** (notes per second, see Table 3 and Figure 4). Compared with *X. jinggangensis* and *X. cheni* **sp. nov.**, *X. lini* **sp. nov.** has a unique pattern of calls, in each single call the inter-note intervals gradually increase as the call comes to an end (Table 3 and Figure 4).

**Table 3:** Vocalization parameters of six *Xenophrys* species in southeast China.

Species	call duration	notes per call	notes per second	note duration	inter-note intervals	high frequency	low frequency	frequency band-width
**(A)** *X. cheni* **sp. nov.**	9.113±2.513	9.222±2.350	1.125±0.127	0.143±0.008	0.953±0.045	4041±38.66	2799±22.17	1242±48.62
3	9	9	9	88	77	88	88	88
**(B)** *X. lini* **sp. nov.**	3.063±0.210	10.167±0.793	3.310±0.101	0.106±0.001	0.225±0.006	3899±22.37	3006±9.43	893±21.89
6	18	18	18	164	147	164	164	164
**(C)** *X. jinggangensis*	4.983±0.668	26.080±3.727	5.704±0.268	0.077±0.001	0.127±0.003	3879±8.61	2788±7.37	1091±10.38
4	25	25	25	420	405	420	420	420
**(D)** *X. huangshanensis*	8.515±1.312	35.000±5.568	4.102±0.026	0.086±0.001	0.165±0.005	4044±21.40	2945±27.00	1099±28.90
3	3	3	3	42	41	42	42	42
**(E)** *X. boettgeri*	4.937±0.725	25.333±5.696	4.988±0.500	0.054±0.001	0.215±0.021	3959±21.14	3058±13.31	901±13.29
3	3	3	3	76	73	76	76	76
**(F)** *X. kuatunensis*	12.233±6.824	11.250±5.921	0.969±0.047	0.208±0.003	0.970±0.021	4112±35.30	2998±14.06	1114±44.09
2	4	4	4	45	41	45	45	45

The alphabetical order corresponds to the order in Figure 4. The sample size, mean value, and standard deviation are given for each parameter.

The Kruskal–Wallis test of the three individual-independent vocalization parameters (call duration, notes per call, and notes per second) showed significant differences both between the first three sympatric species in Luoxiao Mountains or across all six *Xenophrys* species studied (*p*<0.05 for each parameter of the groups). The test on the five call-independent vocalization parameters (note duration, inter-notes interval, high frequency, low frequency, and frequency band-width) also revealed significant differences within the three sympatric species and across the six *Xenophrys* species recorded (Kruskal-Wallis test, *p*<0.05 for each parameter and groups)

### Morphological comparisons

Specimens examined for morphometric analysis are listed in Table 4 and other specimens for morphological comparisons are listed in Appendix S2. We compared the two new *Xenophrys* species with their 46 known congeners. Of these 46 species, 22 (body-length >45 mm) were larger than the two new species (body-length <40 mm). Of the remaining 23 species, the two new species can be distinguished from 11 of them (*X. ancrae*, *X. jinggangensis*, *X. daweimontis*, *X. oropedion*, *X. pachyproctus*, *X. palpebralespinosa*, *X. parallela*, *X. parva*, *X. serchhipii*, *X. zhangi*, and *X. zunhebotoensis*) by the absence of vomerine teeth; the two new species differ from nine of them (absent horn-like tubercle in *X. binchuanensis*, *X. pachyproctus*, *X. wawuensis*, *X. wuliangshanensis*, *X. wushanensis* and *X. zhangi*; present large horn-like tubercle in *X. jinggangensis*, *X. palpebralespinosa* and *X. parallela*) because they have a small horn-like tubercle at edge on the upper eyelid; of the remaining eight species, the two new species differ from six of them by having wide lateral fringes on the toes versus narrow ones in *X. kuatunensis* and absent in *X. baolongensis*, *X. brachykolos*, *X. huangshanensis*, *X. minor* and *X. tuberogranulatus*. The two new species differ from the one remaining species, *X. boettgeri*, by lacking a large black mark covering most of the dorsum and a light region on upper surface of the scapular; the two new species differ from the last remaining species, *X. vegrandis*, by having subarticular tubercles on toes. Further the tongue is deeply notched in *X. cheni* **sp. nov.** and not notched in *X. lini* **sp. nov.** versus feebly notched in *X. vegrandis*. Moreover, *X. lini* **sp. nov.** is significantly larger than *X. cheni* **sp. nov.** in all involved morphometric parameters (Mann-Whitney U tests: all *p*<0.01) except IND, the internasal distance (Mann-Whitney U tests: *p* = 0.063). Details of morphological comparisons between the two new *Xenophrys* species and other known congeners are presented in Appendix S2 and Table S1.

**Table 4:** Measurements (in mm) of the type series of three sympatric *Xenophrys* species in the middle Luoxiao Mountains, southeast China.

Measurements	*X. lini* sp. nov.		*X. cheni* sp. nov.		*X. jinggangensis*	
	Male (N = 20)	Female (N = 4)	Male (N = 15)	Female (N = 3)	Male (N = 2)	Female (N = 3)
SVL	34.1–39.7 (36.7±1.72)	37.0–39.9 (38.1±1.15)	26.2–29.5 (27.6±1.04)	31.8–34.1 (32.9±1.15)	35.1–36.7 (35.9)	38.4–41.6 (39.7±1.67)
HDL	11.3–13.2 (12.3±0.55)	12.2–13.6 (13.1±0.59)	9.7–10.6 (10.1±0.25)	11.2–12.0 (11.6±0.40)	11.4–12.0 (11.7)	12.2–13.4 (12.9±0.60)
HDW	11.3–13.2 (12.4±0.47)	11.6–13.8 (13.1±0.91)	9.7–10.9 (10.4±0.35)	11.2–12.2 (11.7±0.50)	11.8–11.9 (11.8)	12.2–13.8 (12.9±0.78)
SNT	3.6–4.5 (4.2±0.26)	4.1–4.7 (4.5±0.0.22)	3.3–4.3 (3.7±0.28)	3.9–4.5 (4.2±0.30)	3.4–3.6 (3.5)	4.1–4.6 (4.4±0.24)
IND	3.3–4.2 (3.7±0.26)	3.5–3.9 (3.8±0.24)	3.2–3.6 (3.5±0.13)	3.5–3.8 (3.6±0.15)	3.3–3.5 (3.4)	4.0–4.6 (4.2±0.36)
IOD	3.3–3.8 (3.6±0.16)	3.6–4.1 (3.8±0.22)	2.6–3.4 (2.9±0.24)	2.8–4.3 (3.3±0.84)	3.3–3.4 (3.4)	3.6–4.0 (3.8±0.19)
EYE	3.9–4.8 (4.4±0.27)	4.3–4.4 (4.4±0.05)	3.7–4.3 (3.8±0.16)	4.1–4.4 (4.3±0.15)	3.5–3.8 (3.7)	3.8–4.2 (4.0±0.23)
TMP	1.6–2.5 (2.1±0.25)	2.4–2.6 (2.5±0.08)	1.6–2.1 (1.7±0.19)	2.0–2.3 (2.1±0.17)	2.8–2.9 (2.9)	2.9–3.4 (3.1±0.29)
TEY	2.0–3.1 (2.3±0.25)	2.4–3.2 (2.9±0.31)	1.3–2.1 (1.7±0.21)	1.9–3.1 (2.3±0.69)	2.30–2.42 (2.36)	2.50–2.52 (2.51±0.01)
HND	8.0–9.8 (8.6±0.43)	9.2–9.9 (9.4±0.28)	6.6–8.0 (7.4±0.42)	7.7–8.9 (8.2±0.61)	8.9–9.9 (9.4)	10.4–11.5 (11.0±0.54)
RAD	8.0–9.7 (8.6±0.47)	9.1–9.7 (9.3±0.25)	6.3–7.2 (6.7±0.27)	7.5–8.6 (8.0±0.57)	8.2–8.8 (8.5)	9.1–10.1 (9.7±0.53)
TIB	16.3–19.5 (17.8±0.96)	18.5–20.1 (19.2±0.64)	13.7–16.2 (14.8±0.80)	16.5–17.8 (17.1±0.65)	17.2–17. 6 (17.4)	18.6–19.8 (19.0±0.67)
FTL	21.8–27.2 (24.7±1.33)	26.6–27.1 (26.9±0.19)	18.2–22.9 (20.1±1.37)	23.0–25.0 (23.7±1.10)	23.5–25.0 (24.3)	26.1–28.1 (27.1±0.98)
HDL/SVL	0.32–0.36 (0.33±0.01)	0.32–0.37 (0.35±0.02)	0.35–0.40 (0.37±0.01)	0.34–0.36 (0.35±0.01)	0.33	0.32–0.33 (0.32±0.01)
HDW/HDL	0.97–1.07 (1.02±0.03)	0.95–1.03 (1.0±0.03)	1–1.06 (1.02±0.02)	1.0–1.02 (1.01±0.01)	0.99–1.03 (1.01)	0.97–1.03 (1.00±0.03)
SNT/HDL	0.29–0.36 (0.34±0.02)	0.32–0.37 (0.34±0.02)	0.33–0.42 (0.37±0.02)	0.35–0.38 (0.36±0.02)	0.3	0.33–0.34 (0.34±0.01)
SNT/SVL	0.10–0.12 (0.11±0.01)	0.11–0.12 (0.12±0.01)	0.12–0.15 (0.14±0.01)	0.12–0.14 (0.13±0.01)	0.01	0.10–0.11 (0.11±0.01)
IND/HDW	0.26–0.32 (0.29±0.02)	0.25–0.34 (0.29±0.03)	0.32–0.36 (0.33±0.01)	0.30–0.32 (0.31±0.01)	0.28–0.29 (0.29)	0.31–0.33 (0.32±0.01)
IOD/HDW	0.27–0.31 (0.29±0.01)	0.27–0.32 (0.29±0.02)	0.25–0.33 (0.28±0.02)	0.24–0.35 (0.28±0.06)	0.28–0.33 (0.31)	0.28–0.33 (0.3±0.03)
EYE/HDL	0.32–0.39 (0.36±0.02)	0.32–0.35 (0.33±0.01)	0.36–0.42 (0.38±0.02)	0.36–0.38 (0.37±0.01)	0.31–0.32 (0.32)	0.31–0.32 (0.32±0.01)
EYE/SVL	0.11–0.13 (0.12±0.01)	0.11–0.12 (0.11±0.01)	0.13–0.15 (0.14±0.01)	0.13	0.1	0.10–0.11 (0.10±0.01)
TMP/EYE	0.40–0.60 (0.47±0.06)	0.55–0.59 (0.57±0.02)	0.41–0.54 (0.46±0.04)	0.47–0.52 (0.49±0.03)	0.74–0.82 (0.78)	0.75–0.82 (0.78±0.0)
TEY/TMP	0.83–1.44 (1.13±0.16)	0.92–1.29 (1.16±0.15)	0.71–1.13 (0.95±0.12)	0.83–1.55 (1.11±0.39)	0.79–0.86 (0.82)	0.73–0.88 (0.80±0.08)
HND/SVL	0.22–0.25 (0.23±0.01)	0.24–0.26 (0.25±0.01)	0.25–0.28 (0.27±0.01)	0.24–0.26 (0.25±0.01)	0.24–0.28 (0.26)	0.27–0.28 (0.28±0.01)
RAD/SVL	0.22–0.26 (0.24±0.01)	0.24–0.25 (0.24±0.01)	0.23–0.25 (0.24±0.01)	0.23–0.26 (0.24±0.02)	0.22–0.25 (0.24)	0.23–0.26 (0.24±0.02)
TIB/SVL	0.46–0.53 (0.48±0.02)	0.49–0.52 (0.50±0.01)	0.50–0.56 (0.54±0.02)	0.50–0.54 (0.52±0.02)	0.47–0.50 (0.49)	0.48
FTL/SVL	0.64–0.73 (0.67±0.03)	0.68–0.73 (0.71±0.02)	0.66–0.78 (0.73±0.03)	0.68–0.76 (0.72±0.04)	0.64–0.71 (0.68)	0.67–0.71 (0.68±0.02)

The range, mean value, and standard deviation are given for each parameter.

Based on the results of the molecular, vocalization analyses, and morphological comparisons, we describe two new sympatric species from the middle Luoxiao Mountains as below.

### *Xenophrys lini* Wang and Yang sp. nov

urn:lsid:zoobank.org:act:78DE0687-E2F9-42AB-931D-57601FAAA01C

**Figure 5:**
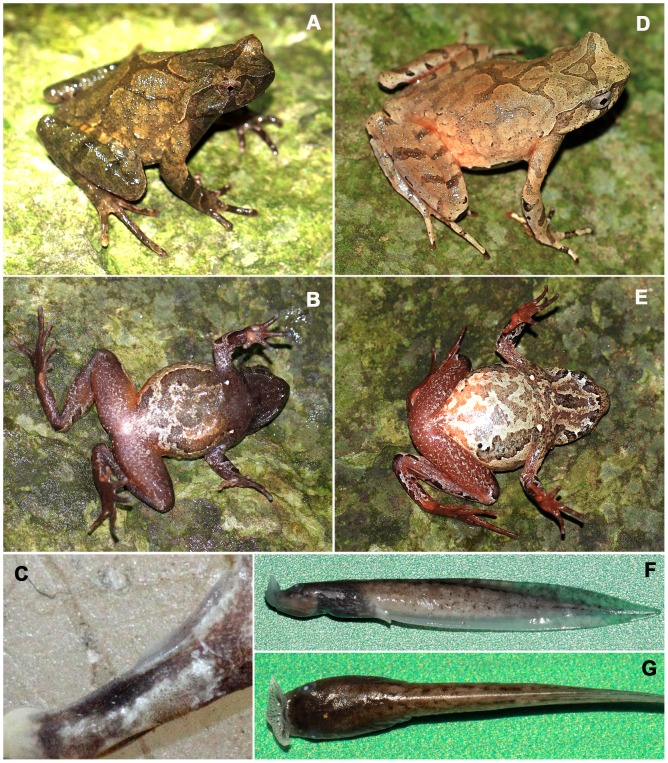
*Xenophrys lini* sp. nov. A. Dorsolateral view of the live adult male *Xenophrys lini* **sp. nov** holotype SYS a001420. B: Ventral view of the live holotype. C: A clump of tiny black nuptial spines on the thumb of the preserved holotype. D: Dorsolateral views of the live adult female paratype SYS r0001423. E: Foot with wide lateral fringes and rudimentary webbings on the toes in paratype SYS a002372. F and G: Lateral and dorsal view of *X. lini* **sp. nov.** tadpole at stage 32^th^ in preservative. Photographed by YYW.

**Holotype:** Adult male, SYS a001420, collected by Jian Zhao (JZ hereafter) on September 19, 2011, from the Bamianshan (26°34′37.97″, 114°06′6.43″E; 1369 m a.s.l.), Mt. Jinggang, Jiangxi Province, China (Figure 5: A, B, and C).

**Paratypes:** Nineteen adult males: SYS a001419 and 001421, collected from the same locality as the holotype at by JZ, Ying-Yong Wang (YYW hereafter) and Run-Lin Li (RLL hereafter) on September 19, 2011; SYS a002381–002386, collected from the same locality as the holotype by JZ on October 6, 2013; SYS a002375–002380, collected from the Jingzhushan (26°29′48.32″N, 114°04′49.21″E; 1150 m a.s.l.) by JZ on October 5, 2013; SYS a002369–002370 and 002372–002374 (Figure 5: E), collected from the Nanfengmian (26°18′40.99″N, 114°02′26.71″E; 1100–1200 m a.s.l.) by YYW and Zu-Yao Liu on October 6, 2013; five adult females: SYS a001417, 001418, 001422, 001423 (Figure 5: D), and 001424, collected by JZ and RLL on September 13, 2011, from the Dabali (26°28′12.43″N, 114°05′7.72″E;1523–1610 m a.s.l.), Mt. Jinggang, Jiangxi Province, China. One juvenile: SYS a002128, collected by JZ and RLL on 21th May, 2013 from Niushiping Village (26°25′19.08″N, 114°02′53.21″E; 1360 m a.s.l.), Taoyuandong Nature Reserve, Hunan Province.

**Other material examined:** Thirty tadpoles, from the Bamianshan and Dabali at 1350–1600 m a.s.l. by JZ and RLL on December 7–9, 2011 (Figure 5: F and G).

**Diagnosis:** *Xenophrys lini* **sp. nov.** is characterized by the combination of the following characters: (**1**) a small-sized species with 34.1–39.7 mm SVL in adult males, 37.0–39.9 mm SVL in adult females; (**2**) head length approximately equal to head width (HDL/HDW ratio 1); (**3**) snout short, obtusely pointed in dorsal view, almost truncate and sloping backward to the mouth in profile, protruding well beyond the margin of the lower jaw; (**4**) vomerine teeth absent; (**5**) margin of the tongue smooth, not notched behind; (**6**) hind limbs elongated, heels overlapping and tibio-tarsal articulation reaching the anterior corner of the eye; (**7**) relative finger length II≤I<IV<III; (**8**) lateral fringes on the digits wide, toes with rudimentary webbing at their bases; (**9**) subarticular tubercle on each digit distinct; (**10**) dorsal skin smooth with scattered granules, usually a few curved weak ridges on back, several tubercles on flanks; (**11**) ventral surface smooth; (**12**) a small horn-like tubercle at the edge of the eyelid; (**13**) supratympanic fold narrow, light colored, curving from the posterior corner of the eye to a level above the insertion of the arm; (**14**) light brown or olive above, a dark interorbital triangular marking and X-shaped dorsal marking bordered with a light edge; (**15**) scattered, tiny, black nuptial spines covering the middle of the dorsal surface of the first finger; (**16**) single vocal sac in males; (**17**) gravid females bear pure yellowish eggs.

**Holotype description:** Adult male, SVL 39.1 mm. Head length approximately equal to head width (HDL/HDW ratio 1.0); snout short (SNT/HDL ratio 0.4, SNT/SVL ratio 0.1), obtusely pointed in dorsal view, almost truncated and sloping backward to the mouth in profile, protruding well beyond the margin of the lower jaw; loreal region vertical, not concave; canthus rostralis well-developed; top of head flat; eye large and convex, eye diameter 35% of head length; pupil vertical; nostril oblique ovoid with low flap of skin laterally; internasal distance larger than interorbital distance; tympanum distinct, TMP/EYE ratio 0.53; tympanum-eye distance great, TEY 2.3 mm, TEY/TMP ratio 0.96; choanae large, ovoid, partly concealed by the maxillary shelves; two vomerine ridges weakly, oblique, posteromedial to choanae, no vomerine teeth; margin of tongue smooth, not notched behind.

Forelimbs moderately slender; radioulna length 23% SVL, hands without webbing, moderately long, 23% of SVL; fingers slender, relative finger length II≤I<IV<III; tips of digits round, slightly dilated; subarticular tubercle distinct at the base of each finger; slight lateral fringes from bases of each fingers to terminal phalanges; two metacarpal tubercles, substantially enlarged. Hind limbs relatively elongated and moderately robust; heels overlapping when the flexed legs are held at right angles to the body axis; tibio-tarsal articulation reaching the anterior corner of the eye, when leg stretched along the side of the body; tibia length 49% of SVL; foot length 66% of SVL; relative toe lengths I<II<V<III<IV; tips of toes round, slightly dilated; subarticular tubercle distinct at the base of each toe; toes with rudimentary webbing at their bases, lateral fringes wide; tarsal fold absent; but as outer lateral fringes on toe V from hough to terminal phalanges; inner metatarsal tubercle ovoid; no outer metatarsal tubercle.

Skin of all upper surfaces and flanks smooth with scattered granules; back with a few curved weak, discontinuous ridges; several tubercles on the flanks and dorsal surface of thighs and tibias; a curved ridge on the upper eyelid, anteriorly starting near canthus rostralis (not in contact), extending backward to the middle of the upper eyelid and bending to the inside, where there is a slightly large, horn-like tubercle; supratympanic fold distinct, narrow, curving posteroventrally from posterior corner of the eye to a level above insertion of the arm; ventral surface smooth; pectoral gland large, round, prominently elevated relative to the ventral surface, closer to the axilla than to the mid-ventral line; single larger femoral gland on rear of thigh; distinct granules on posterior thighs and upper cloaca; cloacal opening unmodified, directed posteriorly, at upper level of thighs.

**Holotype measurements (in mm):** SVL 39.1, HDL 12.7, HDW 13.2, SNT 5.3, IND 4.8, IOD 4.2, EYE 4.5, TMP 2.4, TEY 2.3, HND 8.8, RAD 9.0, FTL 25.9, and TIB 19.3.

**Live holotype coloration:** Olive-brown above with distinct dark brown markings bordered with light edge and obscure markings; a distinct dark triangular marking between the eyes, apex of triangle over occiput; a distinct X-shaped dark marking on the back; a small longitudinal dark stripe on the dorsum of the snout; dorsal surface of the limbs and digits with dark brown transverse bands; side of head with dark brown vertical bars, one from the tip of the snout to behind the nares; one under the eye, one along the supratympanic fold, coving tympanum; supratympanic fold light colored; lower lip black with six vertical white spots; lateral surface of trunk and anterior surface of thighs pinkish near the groin; ventral surface reddish brown, with an obscure longitudinal black streak down the center of the throat, with several white blotches on the belly; ventral surface of the limbs reddish brown with pale gray wormlike marks; palms and soles uniform reddish brown, tip of digits pale white; inner metatarsal tubercle and two metacarpal tubercles pinkish; pectoral and femoral glands white; pupils black; iris dark grey.

**Preserved holotype coloration:** Blackish green above with a black triangle and an X-shaped marking bordered with a distinct light edge line; dorsal surface of limbs and digits with black transverse bands; ventral surface darker brown with white blotches; creamy white replaces the pinkish color in the anterior surface of the thighs and lateral surface of the trunk.

**Tadpole description:** Body slender, oval, flattened above; tail depth slightly greater than body depth, dorsal fin arising behind the origin of the tail, maximum depth near mid-length, tapering gradually to narrow, pointed tip; tail 2.3–2.5 times as long as body length, tail depth 18–20% of tail length in the 28^th^–34^th^ stages; maximum body width 37% of body length in the 34^th^ stage, 35% in the 33^rd^ stage, 30–33% in the 28^th^–31^st^ stages; body depth 30% of body length in the 33^rd^ and 34^th^ stages, 29% in the 32^nd^ stage, 24–25% in the 28^th^–31^st^ stages; eyes large, lateral; nostril dorsolateral, slightly closer to the eyes than to the umbelliform oral disk, rim raised; internasal wider than interorbital; spiracle on left side of the body, closer to the eye than to the end of the body; anal tube extends backward above the ventral fin, opening medial; oral disk terminal, lips expanded and directed upwardly into a typical *Xenophrys* umbelliform oral disk; transverse width of expanded funnel 38–42% of body length in 28^th^–34^th^ stages.

**Coloration in preservative:** All upper and lateral surfaces brown grey with black marks; ventral surface of head red-brown, belly black with pale grey marks, tail and hind limbs creamy white.

**Tadpole measurements:** 34^th^ stage: 13.24 mm SVL, 33.1 mm TaL; 33^nd^ stage: 11.0 mm SVL, 27.5 mm TaL; 32^nd^ stage: 11 mm SVL, 26.6 mm TaL; 31^st^ stage: 11 mm SVL, 27.8 mm TaL; 28^th^ stage: 10–10.2 mm SVL, 23.0–25.0 mm TaL.

**Variation:** Measurements and body proportions of type series are given in Table 4. Color patterns in paratypes are more similar to the holotype, but SYS a001419, 001423, 001424, 002369, 002373, 002375, 002379, 002380, 002382, 002383 and 002385 had a dark triangular marking between the eyes with a light center between the eyes; five female paratypes were light brown above; lower lip black with eight white bands; ventral surface with pinkish, brown, white, and black markings; one longitudinal black streak down the center of the throat, surface of the posterior abdomen near the groin white; several large, black spots on ventral surface of the hind limb, forearm, and wrist.

**Secondary sexual characteristics:** Single vocal sac; scattered, tiny, black nuptial spines cover a circular area at the middle of the dorsal surface of the first finger in two male paratypes and holotype; five gravid female paratypes bear pure yellowish eggs in the oviducts.

**Etymology:** The specific epithet “*lini*” is in honor of late Professor and botanist Ying Lin (1914–2003), vice chancellor (1979–1983) of Nanchang University (Jiangxi Province, China), in recognition of his efforts on biodiversity surveys and research in Mt. Jinggang in the 1970s and 80s.

#### Distribution and biological ecology

Currently, *X. lini* **sp. nov.** is known only from the Bamianshan, Jingzhushan, Nanfengmian Nature Reserve and Dabali, within the range of Mt. Jinggang, Jiangxi Province, and from adjacent Taoyuandong Nature Reserve, Hunan Province which are located in the middle of Luoxiao Mountains, running along the border between the Jiangxi and Hunan Provinces, China. All individuals were found in rushing mountain streams surrounded by moist subtropical evergreen broadleaved forests between elevations of 1100–1610 m (Figure 1-VIII: b, c, e, g and i).

All adult specimens were collected on the 13^th^ and 19^th^ September, 2011 and 5–6^th^ October 2013; males were heard calling day and night during the survey. The male paratype SYS a001421 has mature spermaries in the abdominal cavity, measuring 4.9×2.1 mm in the major and minor axes, respectively. All female paratypes bear pure yellowish mature eggs and atrophic ovary fat. According tadpole stages defined by Gosner [41], the individuals in 28–34^th^ stages were found under rocks in the stream on the 5^th^ December, 2011. Juveniles were collected on 21^th^, May, 2013. Thus, we assume the breeding season of this species likely begins September–October.

### *Xenophrys cheni* Wang and Liu sp. nov

urn:lsid:zoobank.org:act:187855BA-517C-44A4-B436-24716DB98D21

**Figure 6:**
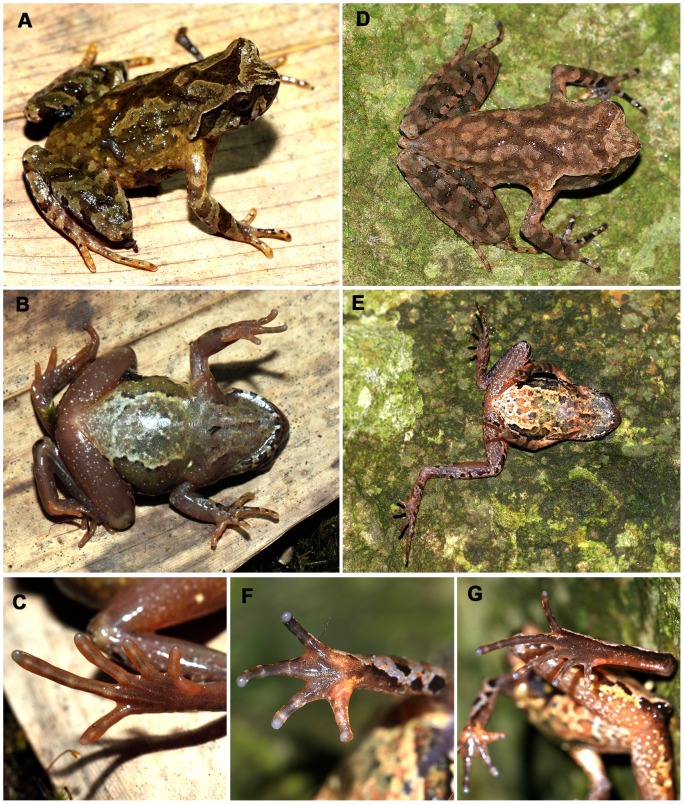
*Xenophrys cheni* sp. nov. A: Dorsolateral view of the live adult male *Xenophrys cheni* **sp. nov.** holotype SYS a001873. B: Ventral view of the live holotype. C: Foot with wide lateral fringes and rudimentary webbing on the toes in the live holotype. D and E: Dorsolateral and ventral views of the live adult female paratype SYS r001429. F and G: Hand and foot of the live paratype SYS r001429. Photographed by YYW and JZ.

**Holotype:** Adult male, SYS a001873 was collected by Jian-Huan Yang (JHY hereafter), RLL, and JZ on 20^th^ July, 2012, from the Jingzhushan (26°29′45.95″N, 114°04′45.66″E; 1210 m a.s.l.), Mt. Jinggang, Jiangxi Province, China (Figure 6: A, B, C).

**Paratypes:** Seventeen paratypes including 14 adult males and three adult females, collected at elevations between 1210–1530 m a.s.l.: SYS a001427 and 001428, adult males, by JZ, YYW, and RLL on September 19^th^, 2011; SYS a001538, adult male, by JZ on April 7^th^, 2012; SYS a001871 and 001872, adult males, by JHY, RLL, and JZ on July 20^th^, 2012; SYS a001429, adult female, by YYW, RLL, and JZ on September 19^th^, 2011 (Figure 6: D, E, F, and G), all above were collected from the same locality as the holotype. The paratypes, SYS a002123 and 002124, adult females, SYS a002125–002127, adult males collected by JZ and RLL on 22th May, 2013, from Lishuzhou Village (26°20′31.33″–26°20′47.7″N, 113°59′01.1″–113°34.5″E; 1480–1530 m a.s.l.), Taoyuandong Nature Reserve, Hunan Province; SYS a002140–002145, adult males, collected by JZ and RLL on 22th May, 2013, from Dayuan Farm (26°23′16.4″N, 114°01′56.2″E; 1480 m a.s.l.), Taoyuandong Nature Reserve, Hunan Province.

**Diagnosis:** *Xenophrys cheni* **sp. nov.** is characterized by the combination of the following characteristics: (**1**) a small-size species with 31.8–34.1 mm SVL in adult females, 26.2–29.5 mm SVL in adult males; (**2**) head length approximately equal to head width (HDL/HDW ratio 1.00–1.06); (**3**) snout short, obtusely rounded in dorsal view, almost truncate, and sloping backward to the mouth in profile, protruding well beyond the margin of the lower jaw; (**4**) vomerine teeth absent; (**5**) margin of tongue notched behind; (**6**) tympanum distinct or indistinct, usually its upper part hidden under the supratympanic fold; (**7**) hind limbs elongated, the heels more overlapping and tibio-tarsal articulation reaching the region between the nostril and tip of snout; (**8**) relative finger length I<II<IV<III; (**9**) lateral fringes on digits wide, toes with rudimentary webbing at their bases; (**10**) subarticular tubercle on each toe indistinct; (**11**) Skin of all upper surfaces and flanks smooth with tubercles, usually forming a dorsolateral tubercle row of parallel to contralateral row, an X-shaped weak ridge between the dorsolateral tubercle rows on dorsum; (**12**) large tubercles arranged in four or five transverse rows on shanks; (**13**) ventral surface smooth; (**14**) a small horn-like tubercle at the edge of the eyelid; (**15**) supratympanic fold swollen, light colored, curving from posterior corner of the eye to a level above the insertion of the arm; (**16**) red-brown or olive-brown above with dark reticular marking on dorsum, dorsal surface of the limbs with dark cross-bars;(**17**) single vocal sac in males.

**Holotype description:** Adult male, SVL 27.2 mm. Head length approximately equal to head width (HDL/HDW ratio 1.0); snout short (SNT/HDL ratio 0.44, SNT/SVL ratio 0.14), obtusely rounded in dorsal view, almost truncate and sloping backward to the mouth in profile, protruding well beyond the margin of the lower jaw; loreal region vertical, not concave; canthus rostralis well-developed; top of head flattened; eye large and convex, eye diameter 38% of head length; pupil vertical; nostril oblique ovoid with low flap of skin laterally; internasal distance larger than the interorbital distance; tympanum (TMP) distinctly rounded, TMP/EYE ratio 0.42; tympanum-eye distance (TEY) 1.5 mm, TEY/TMP ratio 0.94; choanae large, ovoid, partly concealed by the maxillary shelves; two vomerine ridges weakly, oblique, posteromedial to choanae, no vomerine teeth; margin of tongue notched behind.

Forelimbs moderately slender; radioulna length 25% of SVL, hands without webbing, moderately elongated, 25% of SVL; fingers slender, relative finger length I<II<IV<III; tips of fingers round, slightly dilated, narrower than width of terminal phalanges; subarticular tubercles indistinct; markedly enlarged lateral fringes from the bases of fingers to the terminal phalanges; two metacarpal tubercles. Hind limbs relatively elongated and moderately robust; heels overlapped, when the flexed legs are held at right angles to the body axis; tibio-tarsal articulation reaching the region between the nostril and tip of snout, when leg stretched along the side of the body; tibia length 52% of SVL; foot length 71% of SVL; relative toe lengths I<II<V<III<IV; tips of toes round, slightly dilated, narrower than the width of the terminal phalanges; toes with fleshy webs at their bases; subarticular tubercle indistinct; lateral fringes wide; tarsal fold absent; inner metatarsal tubercle ovoid; outer metatarsal tubercle absent.

Skin of all upper surfaces and flanks smooth with tubercles, usually forming a dorsolateral tubercle row of parallel to contralateral row, and an X-shaped weak ridge on the dorsum of body; five large transverse tubercle rows on the dorsal surface of the shanks; ventral surface smooth; a weak horn-like tubercle at the edge of the upper eyelid; supratympanic fold swollen, curving poster-oventrally from the posterior corner of the eye to a level above the insertion of the arm; pectoral gland small, closer to the axilla than to the mid-ventral line; single larger femoral gland on rear of thigh; posterior end of the body protrudes slightly and appears as an arc-shaped swelling, then sloping backward to the ventral body reaching the anal region; cloacal opening unmodified, directed posteriorly at the upper level of the thighs.

**Holotype measurements (in mm):** SVL 27.2, HDL 10.1, HDW 10.1, SNT 3.8, IND 3.6, IOD 3.3, EYE 3.8, TMP 1.6, TEY1.5, HND 6.8, RAD 6.9, FTL 19.4, and TIB 14.1.

**Live holotype coloration:** Olive-brown above with dark reticular markings, including a triangular marking bordered with a light edge between the eyes, apex of triangle over occiput, an X-shaped marking bordered with a light edge on the dorsum of body, five dark transverse bands on the dorsal surface of the thigh, four dark transverse bands on the dorsal surface of the shank; side of head with dark brown vertical bars, one from the tip of the snout to behind the nares; one under the eye, one along the supratympanic fold, coving tympanum; supratympanic fold light colored bordered by a black lower edge; lower lip black with six white bars; lateral surface of trunk of body and anterior surface of the thighs near the groin pinkish; ventral surface of body olive with pinkish and white spots; an obscure longitudinal darker streak down the center of the throat; several white blotches on the belly; the ventrolateral regions covered with olive-green bordered black zigzag lines; ventral surface of limbs darker brown with white spots; palms and soles uniform darker brown, tip of digits pale grey; inner metatarsal tubercle and two metacarpal tubercles pinkish; pectoral and femoral glands white; pupils black; iris dark brown.

**Preserved holotype coloration:** Dorsal surface sallow with darker brown reticular markings; triangular and X-shaped markings on dorsum and transverse bands on limbs and digits distinct; ventral surface yellowish with grey spots; black zigzag lines distinct; creamy-white substitutes the pinkish in the anterior surface of the thighs and lateral surface of the trunk.

**Variation:** Measurements and body proportions of type series are given in Table 4.

Color patterns in 14 male paratypes are more similar to the holotype. In the male paratypes SYS a002124, 002126, 002140, 002141 and 002145, tympanum or its edge is indistinct; in SYS a002126 and 002127, upper part of tympanum hidden under the supratympanic fold; in the female paratypes, red-brown above with brown reticular markings; lower lip black with white spots; gular region and chest black with red and white blotches; posteriorly black fades and becomes marbled with light and dark blotches over the abdomen; in SYS a002123, 002124, 002142, X-shaped markings on back of trunk incomplete or not present.

**Secondary sexual characteristics:** Single vocal sac in all male paratypes and the holotype.

**Etymology:** The specific epithet “*cheni*” is in honor of Mr. Chun-Quan Chen, former director of Mt. Jinggang National Nature Reserve, Jiangxi Province, China, in recognition of his dedication to the biodiversity conservation of Mt. Jinggang.

#### Distribution and biological ecology

Currently, *X. cheni* **sp. nov.** is known from the Jingzhushan, Mt. Jinggang, and adjacent Lishuzhou Village, Dayuan Farm, Taoyuandong Nature Reserve; both located in the middle of Luoxiao Mountains, running along the border between the Jiangxi and Hunan Provinces, China. All individuals were found in mountainous swamps surrounded by moist subtropical evergreen broadleaved forests at elevations of about 1200–1530 m (Figure 1-VIII: c, f and h).

All specimens were collected from April to September, during which all male individuals were calling and bearing dilated spermaries in specimens collected July and September, lacking nuptial spines on the dorsal surface of the first finger; mature ovaries bearing pure yellow eggs and dilated oviducts in the female paratype SYS a001429. Thus, the breeding season of this species is likely from April to September, but no tadpoles were found.

## Discussion

Most cryptic congeners in the genus *Xenophrys* are difficult to distinguish from each other due to the superficial similarities in morphologies: drab colorations, complicated markings and even changeable colorations and skin markings of the same individual under different environmental conditions [9]–[11]. These result in considerable challenges in field identification, which in turn cause ambiguities in taxonomy and distributions [11]. For example, in Luoxiao Mountains covered by our surveys, small sized *Xenophrys* toads occurred were misidentified as either *X. boettgeri*, *X. kuatunensis* or *X. minor* and were documented in the local amphibian checklists [42], [43]. This issue seems ubiquitous throughout the geographical range of *Xenophrys* toads [9]. To solve these problems, extensive sampling with careful and robust diagnosis is necessary in order to unveil the cryptic species diversity of *Xenophrys* toads.

In this study, we characterized the cryptic diversity of *Xenophrys* toads with intensive surveys in the middle range of Luoxiao Mountains, with the realm about 250 km^2^ in southeast China. We discovered two undescribed *Xenophrys* species, namely *Xenophrys lini* **sp.nov.** and *Xenophrys cheni* **sp.nov.** using morphology, molecular genetics and bioacoustics. The two new species, together with *X. jinggangensis*, are sympatric in Luoxiao Mountains but altitudinally and perhaps ecologically isolated (see discussion below). Morphologically these two new species can be reliably distinguished from other known congeners. Although the present genetic analyses are based on only two mitochondrial genes, the genetic differences between the two new species are of a comparable magnitude as other known *Xenophrys* species in southeast China (Table 2). Interestingly, the close phylogenetic relationships between the three sympatric species in Luoxiao Mountains and other known species in southeast China may indicate sign of evolutionary radiation in the region. However, our phylogenies in this study were just partial without including *Xenophrys* species in western China and Himalayas. This encourages further comprehensive phylogenetic analyses with extensive taxa coverage in order to resolve the systematics in *Xenophrys*. Furthermore, although our bioacoustics analysis shows that the advertisement calls of male *Xenophrys* are very similar, consisting of several rather short and repeated notes, the call styles, especially in the aspects of the note frequency ranges, note durations and inter-note intervals show significantly differences between all three sympatric species in Luoxiao Mountains and other three compared congeners.

Based on the present knowledge, the geographic ranges of the two new species as well as *X. jinggangensis* are endemic to several sites in Luoxiao Mountains between Jiangxi and Hunan provinces, China. Luoxiao Mountains are situated in the middle of southeast Chinese subtropical mountain ranges with complex topography and biogeography [44]. It is connected to Nanling Mountains in the south, which is a stronghold for *X. mangshanensis* [7] indicating potential parapatric. It is further paralleled with Huangshan-Tianmu and Yandang-Wuyi-Daiyun Mountains in the east, which harbors *X. kuatunensis, X. huangshanensis* and *X. boettgeri* [7], indicating potential allopatric. In a finer scale, we found that three sympatric *Xenophrys* species in Mt. Jinggang were distributed in different microhabitats, where the altitudes and the characters of the water bodies varied subtly. *X. jinggangensis* was found in slow-moving streams between 700–850 m a.s.l. [10] and *X. lini* **sp. nov.** in rushing streams between 1100–1610 m a.s.l. In contrast, *X. cheni* **sp. nov.** was restricted to swamps at forest edges around 1200–1530 m a.s.l. (Figure 1). Whether this pattern of spatial and ecological segregation in *Xenophrys* toads in Luoxiao Mountains is associated with local adaptation to divergent environments requires further investigation on the diversification mechanisms using ecological [45] and genetic (genomic) approaches [46].

This study further provides a few fresh insights into the taxonomy of *Xenophrys* toads. Most importantly, the identification of two new species may indicate previously underestimated diversity and endemism *Xenophrys* in southeast China [47]. However, the potential spatial or ecological limits of these species are still poorly known. Moreover, the closely related *X. huangshanensis* and *X. boettgeri* show moderate differences in morphology and vocalization and a small genetic distance (0.005) in contrast to the other species studied. The species validation between these two, *X. huangshanensis* and *X. boettgeri*, needs to be revisited. Finally, though we used only the mitochondrial 16s and 12s rRNA as genetic markers for its universal application in molecular analysis in amphibian systematic studies [48], the integrative manner of this study using multiple approaches merits robust species delineation. Regardless, our results provide a first step in the right direction to help resolve previously unrecognized amphibian biodiversity. More spatially extensive sampling ideally combined with habitat characteristics and bioacoustic recordings will be necessary to further understanding the cryptic diversity and diversification of *Xenophrys* toads in the mountain complexes of southeast China.

In conclusion, we show that the two sympatric *Xenophrys* species, *X. lini* **sp. nov.** and *X. cheni* **sp. nov.** have congruent differences in morphology, bioacoustics, genetic and habitats. We are reasonable to treat them as separate species based on the ‘biological species concept’ [49], [50]. Nevertheless, more studies are needed on the distributions, ecology and life history of these locally endemic species, as well as on their conservation status. These efforts are very important given the ongoing declines of amphibians both regionally and globally [47], [51], [52].

## Supporting Information

Appendix S1 **Recordings of typical male advertisement calls of six** ***Xenophrys*** **species.**
(ZIP)

Appendix S2 ***Xenophrys*** **specimens examined in this study.**
(DOCX)

Figure S1 **Bayesian inference and maximum-likelihood phylogenies.** The species *Paramegophrys oshanensis* and *Megophrys nasuta* were chosen as outgroup. Numbers above or below branches are bootstrap values based on 1000 replicates for maximum-likelihood analyses (left, >60 retained) and Bayesian posterior probabilities (right, >0.6 retained).
(DOCX)

Figure S2 **Bayesian inference and maximum-likelihood phylogenies.** The species *Megophrys nasuta* were chosen as outgroup. Numbers above or below branches are bootstrap values based on 1000 replicates for maximum-likelihood analyses (left, >50 retained) and Bayesian posterior probabilities (right, >0.5 retained).
(DOCX)

Table S1 **Morphology comparisons among** ***Xenophrys*** **species.** Characters that differentiate *Xenophrys lini* **sp. nov.** and *X. cheni* **sp. nov.** from all 46 recognized *Xenophrys* species.
(XLSX)
