# Supplementary material for: Morphology, Molecular Genetics, and Bioacoustics Support Two New Sympatric Xenophrys Toads (Amphibia: Anura: Megophryidae) in Southeast China
Source: PLoS One. 2014 Apr 8;9(4):e93075. doi: 10.1371/journal.pone.0093075 (PMC3979665; doi:10.1371/journal.pone.0093075)
Supplement: Appendix S2 — Xenophrys specimens examined in this study. (DOCX) [file pone.0093075.s002.docx]

**Appendix S2.** *Xenophrys* specimens examined in this study.

*Xenophrys boettgeri* (7): SYS a000312, 000315, 000328–000330, 000376, and 000378, Mt. Yangjifeng Nature Reserve, Mt. Wuyi, Guixi City, Jiangxi Province, China, 600–900 m a.s.l., June–August 2008.

*Xenophrys brachykolos* (2): SYS a001502–001503, Hong Kong, February 11, 2012.

*Xenophrys jinggangensis* (5): SYS a001413, 001416 and 001430, adult females; SYS a001414–001415, adult males, Mt. Jinggang at 700–850 m a.s.l., September 13 2011.

*Xenophrys huangshanensis* (10): SYS a001314–001323, Mt. Dazhang, Wuyuan County, Jiangxi Province, China, July 28, 2011.

*Xenophrys kuatunensis* (1): SYSa000241, Mt. Yangjifeng Nature Reserve, Guixi, Jiangxi Province, China, 950 m a.s.l., April 13, 2008.

*Xenophrys mangshanensis* (8): SYS a000288, Danxiashan Geology Park, Renhua County, Guangdong Province, China, 300 m a.s.l., May 18, 2008; SYS a000493, 00494, 00495, 00496, and 00586, Mt. Nanling, Ruyuan County, Guangdong Province, 800–1100 m a.s.l., May 24, 2009 and August 14^th^ 2009; SYS a000996, 000997, Mt. Jiulian Nature Reserve, Liannan County, Jiangxi Province, China, July 23, 2010.

**Morphological comparisons**

Comparative data of *X. lini* **sp. nov.** and *X. cheni* **sp. nov.** with 46 known species of *Xenophrys* were obtained from the literature (Boulenger 1908; Inger & Romer 1961; Taylor 1962; Fei *et al*., 1992; Inger *et al*., 1995; Rao & Yang, 1997; Tian *et al*., 2000; Ohler *et al*., 2002; Inger & Iskandar 2005; Stuart *et al*., 2006; Mathew & Sen 2007; Fei *et al*., 2009; Mo *et al*., 2010; Mahoney, 2011; Wang *et al*., 2012; Mahoney *et al*., 2013) and are presented in Appendix 1 and Table S1.

The two species can be readily distinguished by their smaller body size with 34.1–39.7 mm SVL in adult males and 37.0–39.9 mm SVL in adult females in *X. lini* **sp. nov.,** and 26.2–29.5 mm SVL in adult males and 31.8–34.1 mm SVL in adult females in *X. cheni* **sp. nov.** versus the larger body size (> 45 mm SVL) in the adults in the following 23 congeners: *X. aceras* (Boulenger, 1903), *X. auralensis* (Ohler, Swan & Daltry, 2002), *X. baluensis* (Boulenger, 1899), *X. binglingensis* (Jiang, Fei & Ye, 2009), *X. caudoprocta* (Shen, 1994), *X. damrei* (Mahony, 2011), *X. dringi* (Inger, Stuebing & Tan, 1995), *X. gigantica* (Liu, Hu & Yang, 1960), *X. glandulosa* (Fei, Ye & Huang, 1990), *X. jingdongensis* (Fei & Ye, 1983), *X. lekaguli* (Stuart, Chuaynkern, Chan-ard & Inger, 2006), *X. longipes* (Boulenger, 1886), *X. major* (Boulenger, 1908), *X. mangshanensis*, *X. medogensis* (Fei & Ye, 1983), *X. nankiangensis* (Liu & Hu, 1966), *X. omeimontis*, *X. robusta* (Boulenger, 1908), *X. sangzhiensis* (Jiang, Ye & Fei, 2008), *X. shapingensis* (Liu, 1950), *X. shuichengensis* (Tian, Gu & Sun, 2000), *X. spinata* (Liu & Hu, 1973), and *X. takensis* (Mahony, 2011); by absent versus present vomerine teeth in the following 25 congeners: *X. jinggangensis*, *X. aceras*, *X. ancrae* (Mahoney *et al*., 2013), *X. baluensis* (Boulenger, 1899), *X. caudoprocta*, *X. damrei*, *X. daweimontis* (Rao & Yang, 1997), *X. glandulosa*, *X. jingdongensis*, *X. lekaguli*, *X. longipes*, *X. major*, *X. mangshanensis*, *X. medogensis*, *X.* *omeimontis*, *X. oropedion* (Mahoney *et al*., 2013), *X. pachyproctus* (Huang, 1981), *X. palpebralespinosa* (Bourret, 1937), *X. parallela* (Inger & Iskandar, 2005), *X. parva* (Boulenger, 1893), *X. robusta*, *X. sangzhiensis*, *X. serchhipii* (Mathew & Sen, 2007), *X. takensis*, *X. zhangi* (Ye and Fei, 1992), and *X. zunhebotoensis (*Mathew & Sen, 2007); by having a small horn-like tubercle at the edge of the upper eyelid versus a large one in *X. jingdongensis*, *X. caudoprocta*, *X. palpebralespinosa*, *X. parallela*, *X. shuichengensis*, and versus absent in *X. binchuanensis* (Ye & Fei, 1995), *X. binglingensis*, *X. damrei*, *X. gigantica*, *X. nankiangensis*, *X. pachyproctus*, *X. spinata*, *X. takensis*, *X. wawuensis* (Fei, Jiang & Zheng, 2001), *X. wuliangshanensis* (Ye & Fei, 1995), *X. wushanensis* (Ye & Fei, 1995), and *X. zhangi*; by having wide lateral fringes on the digits versus narrow ones or absent in *X. jinggangensis*, *X. ancrae*, *X. auralensis*, *X. kuatunensis* (Pope, 1929), *X. major*, *X. nankiangensis*, *X. omeimontis*, *X. sangzhiensis*, *X. zhangi*, and versus absent in *X. baolongensis* (Ye, Fei & Xie, 2007), *X. brachykolos* (Inger & Romer, 1961), *X. damrei*, *X. daweimontis*, *X. dringi* (Mahony, 2011), *X. huangshanensis* (Fei & Ye, 2005), *X. lekaguli*, *X. mangshanensis*, *X. medogensis*, *X. minor* (Stejneger, 1926), *X. oropedion*, *X. pachyproctus*, *X. parallela*, *X. parva*, *X. takensis*, *X. tuberogranulatus* (Shen, Mo & Li, 2010), *X. wawuensis*, and *X. wuliangshanensis*, in the male of *X. wushanensis*; by having a small, distinct tympanum versus a large one in *X. jinggangensis* and *X. takensis*, and a hidden tympanum in *X. gigantica*, *X. nankiangensis*, and *X. shapingensis*; further differs from *X. jinggangensis* by having smooth dorsal skin with weak dorsolateral ridges, a smaller tympanum, TMP/EYE ratio 0.36–0.46 in *X. cheni* **sp. nov.** and 0.53–0.60 in *X. lini* **sp. nov.** versus dorsal skin granulated with swollen dorsolateral folds, a larger tympanum, TMP/EYE ratio 0.8 in the latter.

One of the remaining congeners, *X.* *boettgeri* (Boulenger, 1899), differs from *X. cheni* **sp.** **nov.** by having a body size of 34.5‒37.8 mm SVL in males and 39.7‒46.8 mm SVL in females, tongue feebly notched, no subarticular tubercles on toes, a conspicuous, large black mark covers most of the dorsum, upper surface of scapular region light versus smaller body size with 26.2–29.5 mm SVL in males and 31.8–34.1 mm SVL in female, tongue deeply notched, subarticular tubercles on toes indistinct, upper surface of the dorsum red-brown or olive-brown with X-shaped darker marking in the latter; from *X. lini* **sp. nov.** by having tongue feebly notched, a conspicuous, large black mark covered most of the dorsum, upper surface of scapular region light versus not notched, upper surface of the dorsum light brown or olive with a darker X-shaped mark bordered light-edged in the latter. The two new species differ from the last remaining species, *X. vegrandis*, by having subarticular tubercles on toes, further, differ by tongue deeply notched in *X. cheni* **sp. nov.** and not notched in *X. lini* **sp. nov.** versus feebly notched in *X. vegrandis*.

Furthermore, *X. cheni* **sp. nov.** differs from *X. brachykolos* by having relative lengths of fingers I < II< IV < III, legs relatively long, the heels more overlapping and tibio-tarsal articulation reaching the region between the nostril and tip of snout versus IV <II < I < III, legs relatively short, the heels not meeting and tibio-tarsal articulation reaching the shoulder in the latter; from *X. minor* by having smaller body size with 26.2–29.5 mm in males and 31.8–34.1 mm in female, smaller tympanum 0.41–0.54 of eye diameter, tongue notched, indistinct subarticular tubercles on toes versus 32.2–40.5 mm SVL in males and 42–48.2 mm SVL in males, tympanum 0.68 of eye diameter, tongue no notched, absent subarticular tubercles on toes in the latter; from *X. serchhipii* by having relative finger length I < II < IV < III versus 2nd and 4th fingers sub equal in length in *X. serchhipii*; from *X. zunhebotoensis* in that head length is approximately equal to head width versus head length smaller than head width, flank scattered with large tubercles versus flank scattered with small tubercles in latter.

The new species *X. lini* **sp. nov.** further differs from *X.* *brachykolos* by having relative lengths of fingers II≤I< IV < III, legs relatively long, the heels more overlapping and tibio-tarsal articulation reaching the region between the nostril and tip of snout versus IV <II < I < III, legs relatively short, the heels not meeting and tibio-tarsal articulation reaching the shoulder in the latter; from *X. minor* by having a smaller tympanum 0.40–0.60 of eye diameter, tongue no notched, with wide lateral fringes and more extended web on the toes versus the tympanum 0.68 of eye diameter, tongue feebly notched, without lateral fringes and mere rudiment of web in the latter; from *X. serchhipii* by having relative finger length I < II < IV < III versus 2nd and 4th fingers sub equal in length in *X. serchhipii*; from *X. zunhebotoensis* in that head length is approximately equal to head width versus head length smaller than head width in latter.

The new species *X. cheni* **sp. nov.** differs from *X. lini* **sp. nov.** by having significantly smaller body size with 26.2–29.5 mm in males (statistical test) and 31.8–34.1 mm in female (statistical test), tongue notched, relative lengths of fingers I < II< IV < III versus body size with 34.1–39.7 mm SVL in males (statistical test) and 37.0–39.9 mm SVL in females (statistical test), tongue not notched, relative lengths of fingers II ≤ I < IV < III in the latter.

**References**

Boulenger GA (1908) A revision of the Oriental pelobatid batrachians (genus Megalophrys). Proc Zool Soc London, 78: 407-430.

Fei L, Hu SQ, Ye CY, Huang YZ (2009) Fauna Sinica, Amphibia Vol. 2, Anura Ranidae. Science Press, Beijing, 485 pp.

Fei L, Ye CY (1992) Two new species of the Megophrys, Pelobatidae (Amphibia: Anura) from China. Zool Res 13: 5-12.

Frost DR, Grant T, Faivovich J, Bain RH, Haas A, et al. (2006) The amphibian tree of life. Bull Ame Mus Nat His 297: 1-370.

Frost DR (2011) Amphibian Species of the World Version 5.5, an Online Reference. New York, USA: American Museum of Natural History. Accessed 30 September 2013.

Gosner KL (1960) A simplified table for staging Anuran embryos and larvae with notes on identification. Herpetologica16: 183-190.

Inger RF, Iskandar DT (2005) A collection of amphibians from West Sumatra, with description of a new species of Megophrys (Amphibia: Anura). Raff Bull Zool 53: 133-142.

Inger RF, Romer JD (1961) A new Pelobatid frog of the genus Megophrys from Hong Kong. Fieldiana: Zoology 39: 533-538.

Inger RF, Stuebing RB, Lian TF (1995) New species and new records of anurans from Borneo. Raff Bull Zool 43: 115-132.

Mahony S (2011) Two new species of Megophrys Kuhl & van Hasselt (Amphibia: Megophryidae), from western Thailand and southern Cambodia. Zootaxa 2734: 23-39.

Mathew R, Sen N (2007) Description of two new species of Xenophrys (Amphibia: Anura: Megophryidae) from north-east India. Cobra 1: 18-28.

Mo XY, Shen YH, Li HH, WuXS (2010) A new species of Megophrys (Amphibia: Anura: Megophryidae) from the northwestern Hunan Province, China. Curr Zool 56: 432-436.

Ohler A, Swan SR, Daltry JC (2002) A recent survey of the amphibian fauna of the Cardamom Mountains, Southwest Cambodia with descriptions of three new species. Raff Bull Zool 50: 465-481.

Tian YZ, Gu XM, Sun AQ (2000) A new species of Megophrys in China (Amphibia: Pelobatidae). Acta Zootaxon Sin 25: 462-466.

Taylor EH (1962) The Amphibian Fauna of Thailand. Uni Kansas Sci Bull 43: 284-302.
