# Supplementary material for: Morphology, Molecular Genetics, and Bioacoustics Support Two New Sympatric Xenophrys Toads (Amphibia: Anura: Megophryidae) in Southeast China
Source: PLoS One. 2014 Apr 8;9(4):e93075. doi: 10.1371/journal.pone.0093075 (PMC3979665; doi:10.1371/journal.pone.0093075)
Supplement: Figure S1 — Bayesian inference and maximum-likelihood phylogenies. The species Paramegophrys oshanensis and Megophrys nasuta were chosen as outgroup. Numbers above or below branches are bootstrap values based on 1000 replicates for maximum-likelihood analyses (left, >60 retained) and Bayesian posterior probabilities (right, >0.6 retained). (DOCX) [file pone.0093075.s003.docx]

**Figure S1**

***Paramegophrys oshanensis***

***Megophrys nasuta***

**88/0.58**

**78/1.0**

**--/0.99**

**95/0.99**

**87/0.94**

**95/1.0**

**93/--**

**85/0.98**

**61/0.95**

**52/0.55**

**97/0.95**

***Xenophyrs lini* sp.nov**.

***X. cheni* sp.nov**.

***X. jingangensis***

***X. brachykolos***

***X. minor***

***X. boettgeri***

**0.02**

**Figure S2**

**100 /1.0**

**91 /0.97**

**-- /0.54**

**99/1.0**

**98 /0.98**

**90 /0.98**

**100 /1.0**

**90 /1.0**

**-- /0.99**

***Megophrys nasuta***

***X. lini* sp.nov**.

***X. cheni* sp.nov**.

***X. brachykolos***

***X. jingangensis***

***X. boettgeri***

***Xenophyrs minor***

**0.1**
