# Supplementary material for: Morphology, Molecular Genetics, and Bioacoustics Support Two New Sympatric Xenophrys Toads (Amphibia: Anura: Megophryidae) in Southeast China
Source: PLoS One. 2014 Apr 8;9(4):e93075. doi: 10.1371/journal.pone.0093075 (PMC3979665; doi:10.1371/journal.pone.0093075)
Supplement: Figure S2 — Bayesian inference and maximum-likelihood phylogenies. The species Megophrys nasuta were chosen as outgroup. Numbers above or below branches are bootstrap values based on 1000 replicates for maximum-likelihood analyses (left, >50 retained) and Bayesian posterior probabilities (right, >0.5 retained). (DOCX) [file pone.0093075.s004.docx]

**Figure S2**

**100 /1.0**

**91 /0.97**

**-- /0.54**

**99/1.0**

**98 /0.98**

**90 /0.98**

**100 /1.0**

**90 /1.0**

**-- /0.99**

***Megophrys nasuta***

***X. lini* sp.nov**.

***X. cheni* sp.nov**.

***X. brachykolos***

***X. jingangensis***

***X. boettgeri***

***Xenophyrs minor***

**0.1**
